# A Review of Bioactive Release from Nerve Conduits as a Neurotherapeutic Strategy for Neuronal Growth in Peripheral Nerve Injury

**DOI:** 10.1155/2014/132350

**Published:** 2014-07-21

**Authors:** Poornima Ramburrun, Pradeep Kumar, Yahya E. Choonara, Divya Bijukumar, Lisa C. du Toit, Viness Pillay

**Affiliations:** Wits Advanced Drug Delivery Platform Research Unit, Department of Pharmacy and Pharmacology, School of Therapeutic Sciences, Faculty of Health Sciences, University of the Witwatersrand, 7 York Road, Parktown, Johannesburg 2193, South Africa

## Abstract

Peripheral nerve regeneration strategies employ the use of polymeric engineered nerve conduits encompassed with components of a delivery system. This allows for the controlled and sustained release of neurotrophic growth factors for the enhancement of the innate regenerative capacity of the injured nerves. This review article focuses on the delivery of neurotrophic factors (NTFs) and the importance of the parameters that control release kinetics in the delivery of optimal quantities of NTFs for improved therapeutic effect and prevention of dose dumping. Studies utilizing various controlled-release strategies, in attempt to obtain ideal release kinetics, have been reviewed in this paper. Release strategies discussed include affinity-based models, crosslinking techniques, and layer-by-layer technologies. Currently available synthetic hollow nerve conduits, an alternative to the nerve autografts, have proven to be successful in the bridging and regeneration of primarily the short transected nerve gaps in several patient cases. However, current research emphasizes on the development of more advanced nerve conduits able to simulate the effectiveness of the autograft which includes, in particular, the ability to deliver growth factors.

## 1. Introduction

The persistence of peripheral nerve injuries as a challenge has over the years stimulated a significant response in the amount of research produced towards the investigation of strategies to overcome the ordeals of this debilitating clinical condition. The peripheral motor and sensory nerves form an extensive and intricate network encompassing a substantially large area of the body. This extensive placement of nerve tissue makes the peripheral nervous system susceptible to trauma inflicted by external forces to any site of the body [1]. Injuries to peripheral nerves are becoming increasingly common due to frequent incidents of trauma resulting from motor vehicle accidents, fractures, lacerations, crush injuries, and surgical complications that cause direct or indirect nerve compression from oedema and haematomas [2–6]. Peripheral nerve injuries greatly impair the ability to feel normal sensations and exercise muscle movements due to denervation of adjacent tissues and muscles bringing about the loss of sensory and motor function [7]. This results in paralysis, chronic pain, and neuropathies leading to severe disability and a diminished quality of life in those patients who have sustained such injuries [1, 3, 8–12].

Initially, strategies focused on the design of a support structure to perform a similar neuronal function as the gold standard of treatment, a nerve autograft, thereby eradicating the need of obtaining a donor nerve via lengthy or multiple surgical procedures as well as eliminating the concerns of donor tissue availability, resulting morbidities, and additional injuries and scarring [2, 13–18]. A nerve autograft involves the transplantation of a donor nerve across a nerve gap defect. In certain cases, a cable graft is required wherein several donor nerves are attached together to create an appropriate sized graft to fit the recipient nerve. Although nerve autografts have a good success rate in the regeneration of nerve across gaps of less than 10 mm, it becomes less feasible for longer gap defects largely due to the difficulty in extracting sufficient donor nerve tissue and obtaining nerves of appropriate size in diameter to match that of the damaged nerve [16]. Such concerns led to the fabrication and application of artificial synthetic and natural polymeric nerve conduits to span gaps and guide the regrowth of transected nerves by providing a means of structural support and barrier function against the infiltration of scar-forming tissue using hollow polymeric tubing [19–22]. To name a few, commercialised polymer nerve conduits currently on the market and approved for clinical use in humans are NeuroTube, NeuroFlex, NeuroMatrix, NeuroLac, and NeuroGen which are principally composed of collagen with the exception of NeuroTube and NeuroLac which are instead fabricated using polyglycolic acid and poly lactide-co-*ε*-caprolactone, respectively [19, 23–25]. Although they are able to bridge nerve gaps, provide a regenerative and protective environment for damaged nerves, and have been reported to perform therapeutically well in certain cases, their efficacy in nerve regeneration of large nerve gaps does not fully emulate that of an autologous nerve graft. Furthermore, their fairly simple design as a hollow tube does not provide the complete features required for optimal nerve regeneration and functional recovery as listed in Table 1 [19, 26–28]. The deficiency in the success rate of tissue regeneration with these hollow conduits has prompted the development of newer types of nerve conduits [29].

This review article discusses the recent strategies employed for the delivery of growth factors for the application of peripheral nerve regeneration. Characteristics of an ideal nerve conduit necessary for the regeneration of nervous tissue has been briefly mentioned; however, this paper focuses more on the release kinetics of growth factor entrapped polymeric systems. An attempt has been made to highlight the effects of the mechanisms that govern the release of incorporated growth factors from the delivery system as this determines the final dose of growth factor released and its impact on tissue regeneration.

## 2. Modern Development of Nerve Conduits and the Inclusion of Neurotrophic Growth Factors

**Figure 1:**
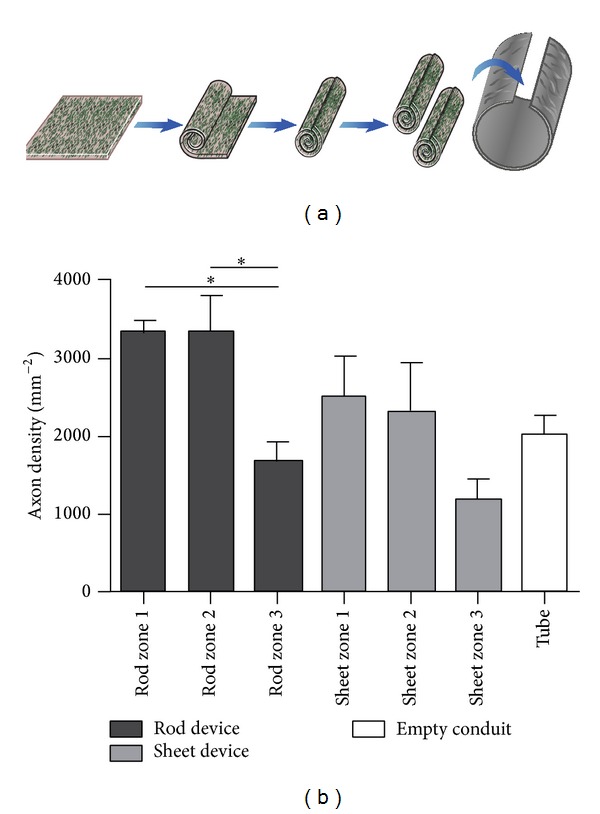
Comparison between the inclusion of rods (act as physical guidance cues) and sheets of a collagen-based engineered neural tissue (EngNT) seeded with Schwann cells within conduits* in vivo*. Flat sheets of EngNT were compared to rods that were made by rolling the sheets, as an approach to delivery within a repair device (a). The different device designs were compared after 4-week recovery in a 5 mm rat sciatic nerve gap model, in terms of the distribution of regeneration within and around the EngNT in cross sections (b). Three zones were compared within each device group—the EngNT (zone 1), a region 25 *μ*m from the EngNT surface (zone 2) and the remaining area within the conduit (zone 3). An empty conduit was also included [36] (reproduced with permission from Elsevier B.V. Ltd., 2013).

Nerve conduits under current development have evolved considerably compared to their hollow polymer tube counterparts in terms of the significant modifications in scaffold design and selection of polymer materials. Despite the major advances and various approaches in the design of nerve conduits and repair strategies, the issue of insufficient functional recovery after peripheral nerve injury remains an obstacle that is yet to be overcome [37–39]. Research in this field of tissue regeneration has focused immensely on the fabrication of nerve conduits able to provide multiple features that may possibly enhance nerve regrowth and restore functional recovery in a smaller time frame as opposed to previous strategies based solely on bridging nerve gap defects [40]. Formerly, research was primarily concentrated on the bridging of nerve gaps and evaluation of the results thereafter with little concern for the determination of the extent of functional recovery achieved and degree of similarity to physiological tissues [30]. Currently, escalating focus is being placed on producing nerve conduits that are able to closely mimic the structure and function of native nerve tissues. This strategy is thought to improve the promotion of nerve regeneration to a level equal to or above that of a nerve autograft [32, 41–43]. To achieve this degree of resemblance to native nerve tissues, numerous physical, chemical, and biological factors have to be taken into consideration when incorporating multiple-functioning components into a nerve conduit [44, 45]. Table 1 lists the pertinent characteristics of a basic peripheral nerve conduit model. The improvement in axonal regeneration when Schwann cells are supplied together with rod-like physical guidance cues in a nerve conduit is shown in Figure 1.

### 2.1. Neurotrophic Factors for Improved Peripheral Nerve Regeneration

While peripheral nerves possess the inherent ability to regenerate and sprout new axons, this innate capacity is often insufficient for the regrowth of an adequately healthy and functional nerve [10, 46]. For the promotion of axonal growth and functional recovery, many studies have concentrated on the effects of sustained-delivery technologies on the enhancement of nerve regeneration. This includes neurotrophic factors within the nerve conduits, particularly in gap defects of 10 mm and larger. The use of neurotrophic factors has been widely studied* in vitro* and* in vivo* and has been proven to enhance nerve regeneration across gaps by enhancing both the rate and quality of nerve regeneration and potentially restoring a marked functional recovery [8, 47–50]. Some leading studies have shown the benefits of utilising combinations of neurotrophic factors that have been incorporated into nerve conduits to determine whether neural regeneration would be further enhanced when compared to the use single agents [51, 52]. Elements which critically influence the regeneration capacity and rate of damaged nerves supplied with exogenous neurotrophic factors (NTFs) are (i) the doses of the neurotrophic factors and their release kinetics at the site of the target tissue and (ii) the effects of initial burst release and whether the employment of single or multiple neurotrophic factors is used to create a synergistic effect on the promotion of nerve growth.

### 2.2. Impediments Associated with the Inclusion of NTFs

The ability of a scaffold-based delivery system to release therapeutically adequate quantities of NTFs and other bioactives is generally influenced by the type of materials used to fabricate the delivery system and the mechanisms that govern the release of NTFs and bioactives to the target tissue as this determines the rate and the quantity of release of the incorporated bioactives. Furthermore, the selection of materials, the method of incorporation of NTFs and bioactives into the delivery system during fabrication, and the end degradation products of the materials used may affect the bioactivity of the NTFs and bioactive agents [53]. The delivery of proteins is considered problematic due to their complex nature and stability [54, 55]. Maintaining bioactivity of proteins is crucial as they are highly prone to degradation and instability by exposure to light, heat, oxygen, agitation, acidic environments, and chemicals [56–58].

## 3. Requirements for Optimal Peripheral Nerve Regeneration

**Figure 2:**
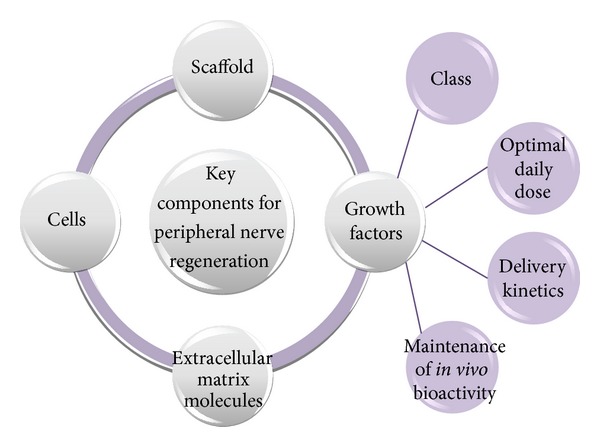
Schematic representation of the basic components required for peripheral nerve regeneration and the factors concerned with the delivery of growth factors.

**Figure 3:**
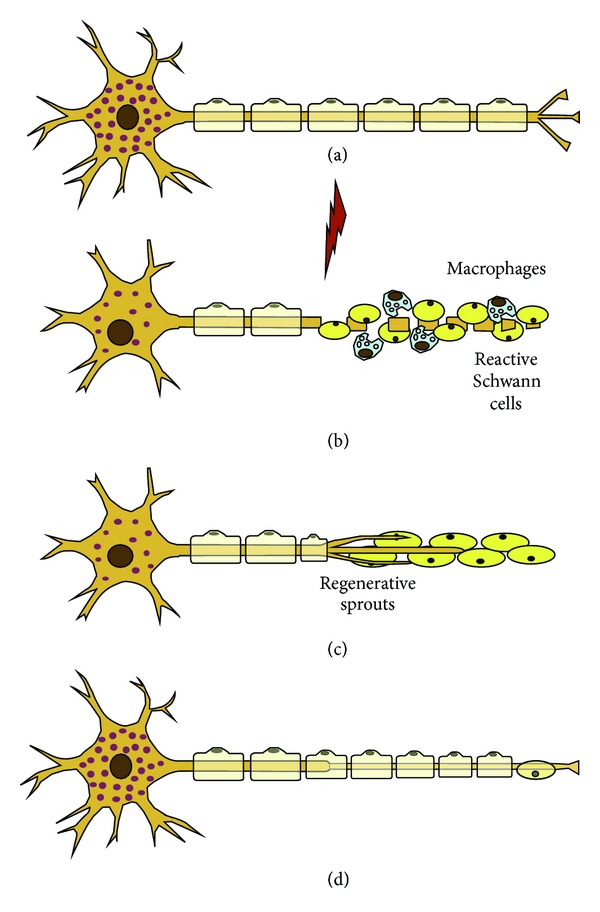
Degeneration and regeneration after peripheral nerve injury. (a) Normal neuron and nerve fiber. (b) Wallerian degeneration. The axotomy results in fragmentation of the distal axon and myelin sheaths. Schwann cells proliferate and macrophages invade the distal nerve segment and phagocytose degrading materials. (c) Schwann cells in the distal segment line up in bands of Büngner. Axonal sprouts advance embedded in the Schwann cells and attracted by gradients of neurotrophic factors. (d) Axonal reconnection with end organs and maturation and remyelination of the nerve fiber [67] (reproduced with permission from Elsevier B.V. Ltd., 2010).

For the successful peripheral nerve regeneration to occur, the injured tissues require four basic components—namely (1) a scaffold, (2) the inclusion of cells, (3) growth factors, and (4) extra cellular material (ECM) molecules—for the neuronal survival, optimal growth, and ultimate regeneration potential (Figure 2). Artificial nerve conduits must be designed in a way that is able to provide a scaffold for the support of growing tissues in conjunction with at least one other component to create a basis for the model of a multifunctioning nerve conduit [2, 3, 59]. By utilizing elements such as scaffold design, growth factor delivery, supply of extracellular matrix proteins, and a substrate or matrix for the attachment of growing cells, the promotion of axonal regeneration and functional recovery can be significantly improved. These features are required for neural cell proliferation and maintenance of cell shape in addition to guiding and provision of mechanical strength to developing axons [60–62]. The implementation of these components into a nerve conduit delivery system will enable a degree of control over the length and processes of the Wallerian degeneration phase [14, 24, 63]. Wallerian degeneration is a term describing the rapid breakdown of axons and myelin sheaths after injury to neural tissues (Figure 3) [64]. Although it is a process triggered for the creation of a microenvironment supportive for nerve regeneration, it additionally initiates the onset of inflammation and intensification of pain [9, 24, 45, 65–68].

## 4. Bioactive Release Kinetics and Its Effects on Optimal Dosing of Growth Factors

In regard to peripheral nerve regeneration, the release kinetics of the selected delivery system is important particularly with reference to the phenomenon of initial burst release, the quantity of NTFs released, and the pattern of its sustained release thereafter. Burst release, defined as a large and immediate release of bioactives and drug from a delivery system within the first 24 hours of placement into dissolution media or biological fluids, can result in overdose effects and dose dumping of the therapeutic agents [69]. Factors which need to be modified to achieve desirable release kinetics of growth factors are represented in Figure 2. Inappropriate quantities of NTFs released from a nerve conduit or other incorporated delivery systems significantly affect the potential of axonal regeneration. The release of high doses resulting from marked or uncontrolled initial burst release may hinder axonal sprouting due to a reduction in the extent of affinity binding for the receptor sites since a lower therapeutic effect will then be achieved in the target tissues [70]. It has previously been noted that suboptimal doses of NTFs evidently does not elicit an adequate effect on the regeneration of nerves but too high doses are nonbeneficial as they may hinder axonal growth due to the downregulation of TrkA and loss of affinity of TrkA to the growth factors, such as nerve growth factor (NGF), in order to initiate a growth response [4, 34, 71, 72].

A study by Conti and coworkers, 2004, proved that high doses of NGF had an inhibitory effect on the neurite growth in dorsal root ganglia (DRG) explanted from wild-type and knockout mice [34]. It was noted that DRG cultures exposed to the low dose of a 5 ng/mL NGF solution exhibited neurite extension of 482 *μ*m in 24 hours whereas an increased NGF solution of 200 ng/mL only supported neurite outgrowth to 173 *μ*m over 24 hours. However, another study using a heparin-immobilization based delivery system for NGF showed that doses of 20 ng/mL and 50 ng/mL elicited greater axonal regeneration and fiber density compared to 5 ng/mL dose across a 17 mm gap in the rat sciatic nerve [73]. Animal studies conducted on sensorimotor and behavioural recovery by Kemp and coworkers, 2011, showed that NGF concentrations of 800 pg/*μ*L provided optimum axonal growth in the early stages of peripheral nerve regeneration compared to animals receiving twice the NGF concentration. However, long-term analysis of peripheral nerve regeneration revealed that animals receiving 80 ng/day for three weeks displayed improved behavioural recovery [4].

Further research is required to evaluate the role of delivery systems, release mechanisms, and release kinetics on the optimal delivery of NTFs in the most beneficial doses for enhanced axonal sprouting. Such factors must be tailored to deliver NTF doses that correspond to the regeneration rate of the injured tissues. Furthermore, understanding the mechanisms behind the phenomenon of initial burst release and factors controlling the characteristics of sustained release profiles will allow researchers to gain a deeper insight and knowledge on modification techniques to regulate the delivery of incorporated bioactives. This enables the rational selection of bioactive agents in the correct doses to be employed in the design of nerve conduits and prevent the economical and therapeutic waste of these rather expensive proteins [69].

### 4.1. Affinity-Based Delivery Systems for the Sustained Release of Glial-Derived Neurotrophic Factor (GDNF) and Nerve Growth Factor (NGF)

Several researchers have focused on* in vitro *and* in vivo* studies utilising heparin-containing affinity-based delivery systems for the sustained release of growth factors in peripheral nerve and spinal cord injuries [73–78]. The release of growth factors in affinity-based delivery systems is controlled by a cell-based degradation mechanism of the matrix into which the growth factor is immobilised as opposed to passive diffusion-based release from biodegradable polymers [73, 74]. In a representative study, a fibrin-based matrix incorporated with a heparin-binding peptide, heparin, and NGF for potential use in nerve conduits was developed to achieve prolonged release of NGF in addition to protecting the bioactive protein from degradation. The release mechanism of the delivery system was designed to release bioactives in response to cellular activities during regeneration via enzymatic factors using a cross-linked heparin-binding peptide to immobilise heparin into the fibrin matrix [74]. Interestingly, the immobilised heparin-conjugated protein was able to slow the diffusion-controlled release of NGF from the fibrin matrix providing a sustained release of growth factor and minimised initial burst release [74]. An earlier study by Sakiyama-Elbert and Hubbell, 2000, involving the development of growth factor-heparin-peptide complexes bound within a fibrin matrix, confirmed satisfactory release of basic fibroblast growth factor through analysis of neurite extension in DRG cultures [75].

**Figure 4:**
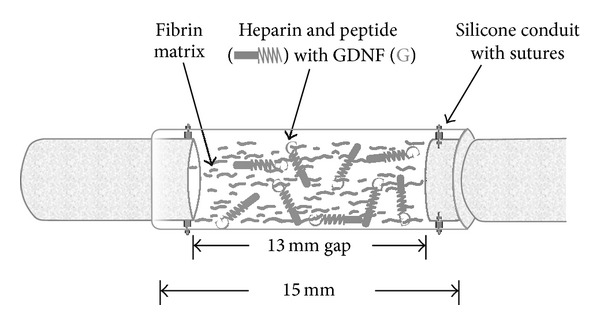
Schematic representation of surgical implantation of nerve guidance conduit containing the affinity-based delivery system. A 13 mm nerve gap was repaired with a 15 mm silicone conduit containing fibrin matrices with or without delivery system and growth factor and sutured to the transected proximal and distal stumps, incorporating 1 mm of nerve on either end. The delivery system consisted of a bidomain peptide crosslinked into the fibrin matrix at one domain while the other binds heparin by electrostatic interactions. The growth factor can then bind to the bound heparin, creating a matrix-bound, nondiffusible complex, which can be retained for cell mediated degradation of the fibrin matrix [77] (reproduced with permission from Elsevier B.V. Ltd., 2009).

Wood and coworkers, 2009, investigated the effectiveness of silicone conduits containing the fibrin immobilized heparin-neurotrophic factor (GDNF and NGF) conjugates on nerve regeneration across a 13 mm sciatic nerve gap (Figure 4) [77]. The GDNF-containing delivery system showed better results in terms of the myelinated fiber count and nerve fiber density compared to the NGF-containing delivery system; however, neither was superior in performance to the nerve isograft. Using the same heparin affinity-based delivery system, the release of NGF exceeded the nerve regenerative effects of the isograft* in vivo* across a 13 mm gap. In another study, Taylor and coworkers, 2004, demonstrated that the ratio of heparin to growth factor can be modified by increasing the heparin content to achieve a sustained and well-defined zero-order release of neurotrophin-3 (NT-3) over 14 days for the repair of spinal cord injuries [78]. Changing the concentration of heparin varies the number of available binding sites to the incorporated growth factors and as a result the release rate of growth factors can be modulated by consequently increasing its retention time within the fibrin matrix [49].

The studies discussed above confirm that affinity-based delivery systems are efficient in promoting neural regeneration as the release rate is allowed to be modulated by the regenerating cells. The neurotrophic factors complexed to peptide-bound heparin form a nondiffusible protein complex allowing it to be restricted to and diffuse out of the matrix system at a slowed rate [77]. In addition, the proteolytic cleavage of the growth factors can be prevented in this manner thereby providing a method for reducing the potential loss of as well as increasing the bioavailability of the bioactives at the site of injury [25, 77]. Affinity-based delivery systems can be used to immobilise a number of growth factors into scaffolds fabricated from a variety of natural and synthetic polymers making it suitable for many tissue engineering applications [79]. Chu and coworkers, 2011, designed a polycation-heparin complex to control the delivery of growth factors over 30 days. The polycation, poly (ethylene argininyl aspartate diglyceride) (PEAD), improved loading efficiency and bioactivity of the growth factors while providing a linear mode of release kinetics [80]. Further modifications to the initial affinity-based approach have resulted in the development of photocrosslinked heparin-alginate, methylcellulose-SH3, and PEG-heparin hydrogels for the controlled and prolonged release of growth factors for bone repair, spinal cord injuries, wound-healing, and vascular therapies [81–83].

Minor burst release effects, reported in these studies, could be attributed to a passive diffusion mechanism. The degree of burst release is dependent on the concentration gradient that exists between the NGF rich matrix and the regenerating tissues. As this concentration gradient is brought into equilibrium by the slowly diffusing NGF from the fibrin matrix, the amount of NGF released is reduced and the release kinetics of the system becomes more uniform until a near zero-order release behaviour is observed. Although passive diffusion may be minimally controlled by external factors, the diffusion rate can be substantially decreased by the crosslinking action of the peptide and immobilization of the heparin into the fibrin matrix which may be considered as the rate-limiting step for the passive release process of the growth factors [8, 74]. Hence, the affinity-based technique of protein delivery has been shown to provide a sustained delivery of bioactives by remarkably slowing down the bioactive release processes which can be tailored by increasing the ratio of bound heparin to NTFs to delay the release of the heparin-binding NTFs [84].

### 4.2. NTF-Loaded Crosslinked Polymer Conduits

Crosslinked polymer conduits offer a relatively simple approach for the entrapment of NTFs. The nature of crosslinking, either physical or chemical, affects the release behaviour of the incorporated bioactive molecules by influencing the water uptake, swelling, and erosion characteristics of the polymer material. Crosslinking not only affects the polymer but also can have an effect on the entrapment of NTFs depending on the crosslinking agent used, its mechanism of action, and the chemical structure of the NTFs. Effects of crosslinking on NTFs comprise mainly interaction of specific chemical moieties between the crosslinker and the NTF or via the differences in the electrostatic charge. A few studies have shown the effects of ionic and covalent crosslinking on the release of NTFs from nerve conduits and microsphere-loaded NTFs [85–87]. In a study by Madduri and coworkers, 2010, poly (lactic-co-glycolic) acid (PLGA)-coated crosslinked and noncrosslinked collagen tubes were evaluated for* in vitro* release as potential sustained-release peripheral nerve conduits [52]. Collagen tubes, physically crosslinked using a dehydrothermal treatment (DHT), were loaded with equal doses of GDNF and NGF. Exterior coating of the collagen tubes restricted the incorporated NTFs within the collagen tube lumen preventing the escape of the NTFs through the walls of the tube. Reducing the chance of NTF loss via escape or leakage from the conduit ensures that the bioavailability of the loaded dose is not compromised and that the full intact dose becomes available to the damaged tissues over the entire regeneration period [27, 88, 89].

Mechanical strength, one of the factors that determine the degradation properties of the scaffold, was controlled by the induction of crosslinking and PLGA-coatings. Noncrosslinked collagen tubes released high quantities of NTFs within the first 2-3 days whereas the crosslinked collagen tubes were able to minimise burst release to a level that was indistinct. The crosslinking process, achieved through a reaction between the free amino and carboxylic acid groups in collagen, was able to retard the degradation of the collagen tubes in the presence of collagenase [52]. This allowed crosslinked tubes to deliver a sustained-release of NTFs over 30 days whereas noncrosslinked tubes rapidly degraded within 2 days. Regarding the dose of NTFs used, 80 ng of GDNF alone was sufficient for the regeneration of the sciatic nerve across a 10 mm gap within 14 days of implantation of the PLGA-coated crosslinked collagen nerve conduits as opposed to tissue growth in conduits, spanning a similar gap length, without the inclusion of NTFs being noted after 30 days [52, 90–92]. Similarly, genipin-crosslinked chitosan nerve conduits immobilized with NGF developed by Yang and coworkers, 2011, provided a sustained release of NGF over 60 days. A burst release of 2.1 ng/day of NGF was reported during the first 3 days progressing to a steady decrease in NGF release over the following 20 days. The release kinetics stabilized to exhibit zero-order release over the next 40 days with a consistent quantity of 0.22–0.25 ng of NGF being released daily. Likewise, poly caprolactone (PCL) tubes internally lined with a coat of genipin-crosslinked gelatin were able to deliver NGF over 60 days in a zero-order manner delivering less than 1 ng daily.* In vivo* studies showed that the crosslinked tubes produced the regeneration of more axons compared to noncrosslinked tubes and the autografts [87].

**Figure 5:**
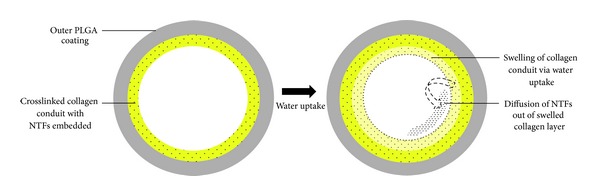
Representation of the design and release from the collagen crosslinked conduits via water uptake and swelling initiated diffusion.

The mechanism of genipin crosslinking involves a reaction with amine groups which in this case was present in both the polymer as well as the NTF. The use of genipin as a crosslinker enhanced the ability of the delivery system in mitigating burst release and obtaining a zero-order release profile by altering the swelling and degradation properties of chitosan in addition to effectively trapping NGF molecules within the polymer network via its reaction with –NH_2_ groups [86, 87, 93, 94]. In the studies described, the primary mechanism controlling the release of NTFs is polymer degradation along with passive-diffusion during the initial release phase. Crosslinking is effective in modifying water-uptake ability, swelling, and degradation processes to control the release of bioactives and minimise burst release to achieve sustained and prolonged delivery. Burst release results from swelling of the conduit walls initiated by water uptake into the polymer chains. Swelling produces the formation of a hydration layer which facilitates the transport of bioactives via diffusion which occurs spontaneously and over which little control can be exerted (Figure 5). This hydration layer creates a diffusion pathway for the entrapped bioactives. With time the hydration layers increase as the polymer swells thereby lengthening the pathway of diffusion that the bioactive molecule must cross before it is finally released into the conduit lumen. This mechanism brings about a decrease in release rates over time providing zero-order release kinetics [95]. Combined with slower degradation rates, smaller quantities of bioactives can be delivered over a prolonged time whereas rapid degradation releases large amounts of bioactives in a shorter time.

### 4.3. Microsphere Technologies for the Delivery of NTFs

**Figure 6:**
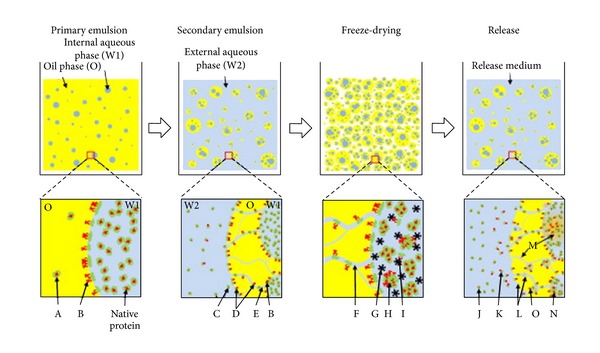
Typical W/O/W double emulsion method to prepare microspheres containing protein drug (upper panel) and microscopic events during fabrication process (lower panel). The sequence of fabrication is primary emulsion, secondary emulsion, solvent extraction/evaporation (not shown), freeze-drying, and drug release test. With negligible partition of protein into oil phase (A), the organic solvent-water interface during W1/O emulsion results in protein denaturation (B). During generation of secondary emulsion, water channels connecting internal (W1) and external (W2) aqueous phases (E) allow proteins to escape from droplets (C) and provide more chances of protein denaturation by increased surface area of the oil-water interface (D). The water channels become pores (F) of microspheres hardened by freeze-drying. Ice crystal (G) is known to provide a hazardous condition inducing protein denaturation (I). Irreversible aggregation (H) between protein molecules can be formed if stabilizer or cryoprotectant is not added. Normally, microspheres made by double emulsion have a broad range of particle size distribution as well as different protein amount in each microparticle. In a release test, a burst release of protein at the initial period (<24 h) is mostly due to the protein release (K) from the proteinaceous film on the particle surface (D). With time, proteins are release from particles (J) by diffusion and degradation (L) of polymer (e.g., PLGA). Microparticle degradation cumulates acidic products inside particles (M), which further facilitates protein denaturation (N). Protein adsorption on hydrophobic polymer surface (O) often leads to incomplete release of protein drugs [100] (reproduced with permission from Elsevier B.V. Ltd., 2010).

Microspheres, usually fabricated from polymeric materials, are spherically shaped particles with diameters in the micrometre range and well known for their capability to deliver drugs and bioactive molecules. Microspheres are frequently used for NTF encapsulation employing different methods of fabrication involving the use of ionic and covalent crosslinkers, double emulsion methods, and spray drying to evaluate the varying degrees of crosslinking, effects on swelling, encapsulation efficiencies, and degradation characteristics as the factors controlling the release of incorporated proteins [96–101]. Several studies have shown promising results in the ability of microspheres to deliver sustained and constant quantities of bioactives as they can effectively be combined with more than one delivery system to control the release kinetics [50, 102, 103]. Careful selection of a suitable method for microsphere preparation is required as the different techniques affect the final outcome on encapsulation efficiency, release kinetics, and preservation of bioactivity of the growth factors (Figure 6) [100].

### 4.4. Crosslinked Chitosan Microspheres

Chitosan microspheres have been widely applied for protein delivery applications with various degrees of success in achieving sustained zero-order release kinetics for periods ranging from 3 days to over 2 months using growth factors, hormones, and bovine serum albumin as model proteins [104–108]. Sinha and coworkers, 2004, extensively reviewed the applications of chitosan microspheres for various categories of drugs while stating the favourable degradation, biocompatibility and hydrophilic characteristics, and simpler processing procedures that chitosan offers [109].

For the application of peripheral nerve injuries, chitosan-PCL microspheres for the delivery of GDNF were expected to release therapeutically sufficient quantities of GDNF in a controlled manner. Having a brush-like chain structure, the authors proposed that the PCL side-chains could act as hooked branches for the entrapment of GDNF by increasing the potential of protein entanglement onto the PCL chains. Depending on the quantities of chitosan, PCL, and the crosslinker used, release profiles varied from near first-order to zero-order kinetics over 49 days. Reduced burst release and zero-order kinetics resulted from the release mechanisms, swelling, diffusion, and degradation, being primarily governed by the increasing amounts of PCL and genipin (crosslinker) used in the microsphere formulations [104].

A study by Zeng and coworkers, 2011, investigated the effects of crosslinking on bioactive release and encapsulation efficiency of NGF-loaded microspheres and obtained similar results to studies described above. Chitosan microspheres were ionically crosslinked using various concentrations of sodium tripolyphosphate (STPP) to control burst release and provide a slow and sustained release of NTF over 7 days. Increase in STPP concentration resulted in a lower NGF encapsulation efficiency but showed a reduction in the initial burst release and offered slow and sustained release of NGF. This is attributed to the high crosslinking density obtained, when STPP concentrations are increased, which prevented swelling of the microspheres and inhibited the rate and reduced the quantity at which NGF is released. STPP of concentrations 1%, 5%, and 10% produced microspheres that exhibited burst release of 45.5%, 24.6%, and 18.4%, respectively, of the total encapsulated dose within 12 hours. The release of NGF from the crosslinked chitosan microspheres occurred in 3 stages: (1) a rapid burst release triggered by swelling, (2) a slower diffusion-based release through pores and channels within the microspheres, and (3) a further slowed release phase dominated by erosion-based release through chitosan biodegradation or in combination with diffusion of NGF [110].

**Figure 7:**
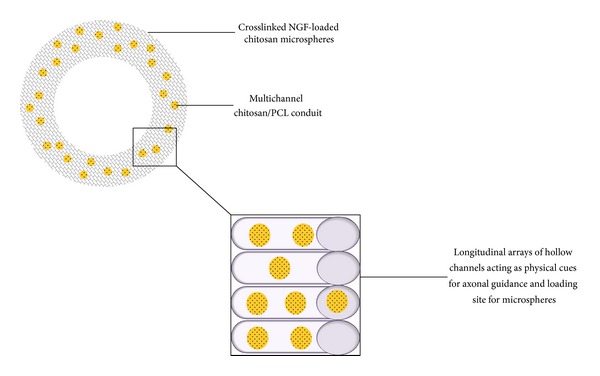
Representation of the internal design and configuration of the multichannel conduits providing physical cues for enhanced regeneration.

Multichannel chitosan-PCL nerve conduits were fabricated to house the microsphere delivery system [111]. The design of the conduit was such that it resulted in longitudinal arrays of hollow cylindrical channels running across the entire length of the tube (Figure 7). In addition to the delivery of NGF the provision of physical guidance cues in the form of these longitudinal channels furthers the promotion of axonal regeneration and accurate direction of growth [15, 112]. The NGF-loaded microspheres embedded into these channels were able to significantly retard the release of NGF in comparison to microspheres alone. The authors proposed that the release behaviour and quantity of the microsphere-encapsulated NGF can be independently controlled by changing the initial loading dose of NGF in the microspheres. To explore this concept, crosslinked chitosan microspheres loaded with initial NGF doses of 5 and 10 ng/mg microsphere were studied* in vitro* over 60 days for differences in release kinetics and quantity of NGF released [111]. There was no significant difference in the burst release and cumulative release of NGF between the high and low loading doses. Both types of microspheres exhibited burst release of approximately 10% and over 60 day study period almost all of the loaded NGF had been released. Despite similar release profiles in terms of percentage cumulative release, it is clear that the higher NGF-loaded microspheres will release a greater amount of growth factor compared to the microspheres loaded with a low initial dose as amount of growth factor present is increased [111].

Crosslinking effects, similar to those described in the study by Zeng and coworkers, 2011, were observed by Liao and coworkers, 2013. Increasing STPP ratios ranging from 1 to 6 of crosslinker : chitosan solution resulted in significantly inhibited swelling of the microspheres by 116% to 70% which controlled swelling to restrain diffusion-based burst release. However, in contrast to the previous reports [110, 111] the encapsulation efficiency was directly proportional to the crosslinker ratios.

### 4.5. NTF-Loaded PLGA Microspheres

**Figure 8:**
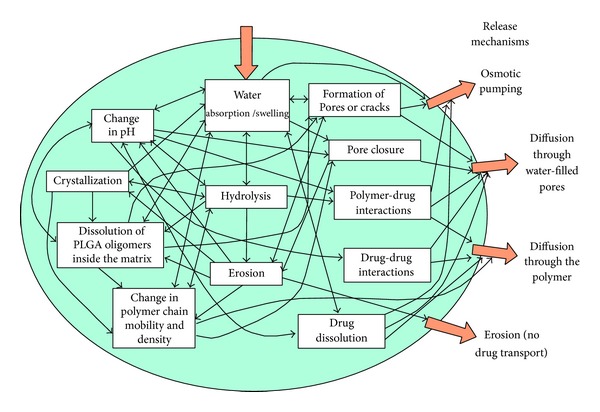
The complex picture of physicochemical processes taking place within PLGA matrices, leading to drug release. The influence of processes on drug release and on other processes is illustrated by arrows. Note that some arrows point in both directions [120] (reproduced with permission from Elsevier B.V. Ltd., 2011).

PLGA microspheres have been extensively investigated for the encapsulation and controlled release of various proteins [100, 113–118]. NGF-loaded PLGA microspheres fabricated by Péan and coworkers, 1998, for injectable administration to the brain also exhibited first-order release kinetics over 12 weeks. Burst release was attributed to the highly porous structure of the microspheres allowing easy penetration of water into the matrix thereby allowing rapid release of NGF via diffusion osmotic pumping (Figure 8) [119, 120]. The prolonged release period was thought to arise from adsorption and entanglement of the protein onto the polymer chains [119]. NGF-encapsulated PLGA microspheres prepared by spray freeze-drying technique achieved a biphasic release over 30 days. The release pattern consisted of an inclining rapid-release phase gradually stabilizing to a zero-order release after medication of the microspheres with zinc carbonate to reduce burst release and enhance sustained release [57]. PLGA microspheres loaded into chitin tubes manufactured by the double emulsion/solvent evaporation technique, releasing BSA as the model protein, provided a first-order release profile over 84 days as investigated by Goraltchouk and coworkers, 2006 [121].

**Figure 9:**
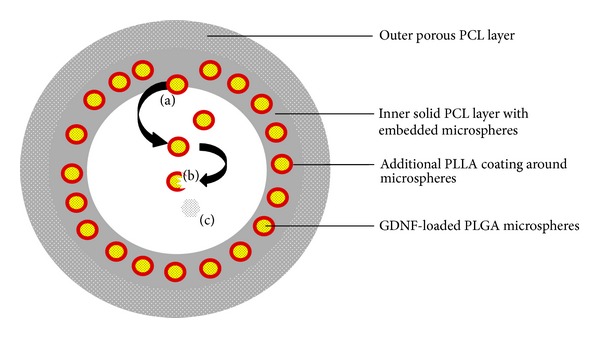
Representation of the double-walled PLGA-PLLA microspheres, (a) release of microspheres from solid PCL layer via degradation of PCL, (b) degradation of microspheres and subsequent release of GDNF, and (c) release of GDNF by diffusion through degradation-formed pores and channels in the microsphere matrix.

Concerning peripheral nerve regeneration, double-walled PLGA-PLLA microspheres loaded with GDNF into the inner walls of a bilayered PCL nerve conduit to achieve sustained delivery for a minimum period of 50 days were developed [122]. The efficacy of the delivery system was evaluated in a rat sciatic nerve model of gap 15 mm over a study period of 6 weeks. Each nerve conduit was loaded with an approximate dose of 95 ng GDNF. The microspheres were embedded into the walls of a PCL conduit which then received another porous layer of pure PCL. This assembly of polymers and microspheres exhibited an initial burst release during the first day followed by a slower near zero-order release over the next 64 days. The release kinetics of the GDNF-loaded microsphere-embedded nerve conduits was able to support the regeneration of blood vessels, intercellular fibers and Schwann cells,* in vivo*, compared to the negative controls [122]. NTF release can further be controlled by a combination of polymer degradation and diffusion processes (Figure 9). The additional layer of PLLA surrounding the GDNF-loaded PLGA microspheres not only increased the time of NTF release by slowing the process of diffusion but also provided a strategy for protection of the GDNF protein by containment of the growth factor within the core of the two-layered microsphere structure. Furthermore, this double-wall of polymers may assist in the prevention of excessive NTF loss as it remains confined within the microsphere core. The inner solid PCL layer, into which the microspheres are embedded, further delays the ultimate release of GDNF as this layer has to erode in order for microspheres to be released into the lumen of the conduit where the microspheres can undergo biodegradation for the diffusion and release of the entrapped GDNF. The outer porous PCL layer of the nerve conduit allows for cellular infiltration and exchange of metabolic substances and nutrients which are vital for the proliferation of physiological tissues [24, 123].

Researchers have proposed that microsphere delivery systems for NGF composed of PLGA exhibited significant initial burst release phases followed by a slow continuous release pattern where the subsequent release of growth factor in the slowed-release phase could be lower than the initial burst release [88]. Furthermore, it is thought that the acidic degradation products of PLGA may cause inactivation of the protein-based NGF creating a concern for the maintenance of NGF stability and ultimate bioactivity [53, 88, 124]. The erosion behaviour of PLGA dominates its ability in controlling release kinetics and inhibiting initial burst release. The interior of PLGA microspheres degrade at a faster rate than the polymer surface on the outside and this bulk-degradation coupled with its autocatalytic mechanism increases the potential for large amounts of entrapped bioactives to be released over a short time frame [125, 126].

### 4.6. Miscellaneous Microsphere Delivery Systems for NTF Delivery

Xu and coworkers, 2002, fabricated polyphosphoester (PPE) microspheres for the delivery of NGF. The PPE polymer, P(DAPG-EOP), used for synthesizing the NGF-loaded microspheres has a phosphate backbone composed of oligomeric D,L-lactide blocks and has been previously shown to exhibit near zero-order release kinetics of drugs used in delivery systems for the application of other disease conditions, by virtue of its collective mechanisms of surface erosion and bulk degradation [127]. PPEs are degraded via hydrolytic and enzymatic cleavage of the phosphate bonds at physiological conditions resulting in the formation of phosphates, alcohols, and diols as the ultimate breakdown products. Hence, it is advantageous in tissue engineering applications as compared to the hydrolysis-induced degradation of PLGA resulting in acidic compounds which may further lower the pH of the entire delivery system and the local surrounding tissues leading to inflammation and inactivation of acid-labile bioactives [120, 127–129]. During the first week, 45% of the loaded NGF was released with an initial burst release of approximately 4%. A slower release phase exhibiting sustained near zero-order release kinetics was observed over the subsequent weeks with an average NGF release rate of 0.5 ng/mg microspheres per day. At the end of the 10-week study period 60% of the total encapsulated NGF was released [88].

*In vivo* studies were performed using the rat sciatic nerve regeneration model where a silicone nerve conduit containing the PPE microspheres was implanted across a 10 mm nerve gap followed by a subsequent study utilizing P(BHET-EOP/TC) conduits as the microsphere carrier. Each conduit, loaded with an NGF dose of 100 ng, was able to, within 2 weeks, promote a positive growth of nerve fibers compared to animals who received conduits filled with a 50 ng/mL NGF saline solution. After 3 months, all animals receiving NGF-loaded microspheres showed a positive muscle reflex, an epineurium-surrounded regenerated nerve cable bridging the 10 mm nerve gap containing myelinated axons, thicker myelin sheaths, and higher fiber densities compared to the controls. The silicone nerve conduits with the NGF-releasing microspheres generated a higher population of axons and fiber density though PPE nerve conduits which prevailed in the regeneration of larger axons and thicker myelin. The authors proposed that the aforementioned NGF delivery strategy would be favourable compared to the nerve autograft and the associated issues of loss function at donor sites, insufficient collection of grafting materials, and the formation of neuromas [88].

GDNF-loaded silk microspheres dispersed throughout a silk conduit were successful in releasing GDNF for Schwann cell migration and nerve tissue proliferation over six weeks [130]. The aqueous solubility of silk offered ideal processing characteristics for the incorporation of growth factors unable to resist harsh fabrication procedures when using polymers requiring acidic and organic solvents [131–133]. Crystalline beta-sheet formation in silk forms physical crosslinks which reinforces the structure of silk imparting enhanced mechanical strength and degradation properties [133, 134]. In another study, photochemically crosslinked collagen microspheres were utilised to preserve NGF bioactivity and with the addition of tween 20, collagen microspheres significantly improved zero-order release of BSA while simultaneously eliminating burst release [85].

## 5. Multiple Layer Strategies and Polyelectrolyte Complexes for the Controlled Release of NTFS

**Figure 10:**
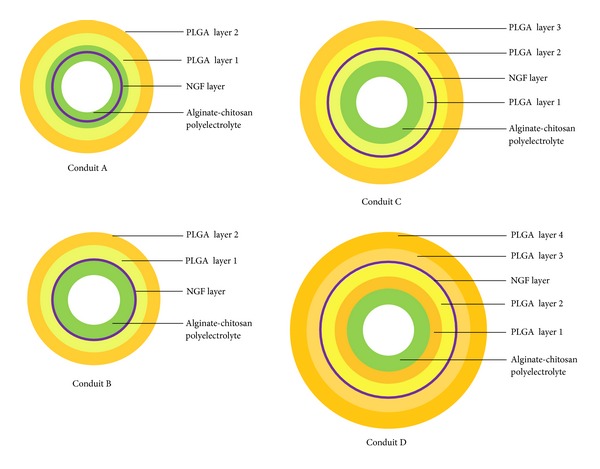
Representation of the different conduits and the various positioning of NGF, alginate-chitosan and PLGA layers.

Another strategy for controlling the release of NTFs is the deposition of bioactive molecules between layers of polymers varying in size, number, material selection, and their ionic charge. In this manner, release kinetics of NTFs can be regulated by changing the quantity of polymeric material in the following ways: (1) by delaying water penetration to hydrate the consequent polymer layers before reaching the NTF layer interface, (2) by increasing the diffusion pathway that must be crossed by the NTF molecules before becoming available to the target tissues, and (3) by extending polymer erosion or degradation before exposure of the NTF layer [95, 122]. As noted in a study by Yang and coworkers, 2011, electrostatic properties can assist with the enhanced entrapment of NTFs via ionic interaction between NTF and crosslinker or NTF and polymer. With layer-by-layer deposition or blending of oppositely charged polymers, various polyelectrolyte complexes (PECs) can be formed for immobilizing NTFs and controlling its release [135, 136]. The outcome on release profiles utilising PECs can be seen in a study conducted by Pfister and coworkers, 2008, using a multiple-layered PLGA-coated system. Hollow nerve conduits were fabricated to control the release of NGF by altering the position of NGF between various concentric layers of PLGA and an alginate-chitosan PEC (Figure 10).

Conduit A, consisting of NGF centrally placed within the alginate-chitosan PEC, was capable of providing a single-phase near-perfect zero-order release kinetics with an indistinct initial burst release. It is thought that the firm entrapment of NGF within the PEC layer was due to electrostatic interaction between the positively charged NGF and the negatively charged alginate [137]. Unlike conduit A, a biphasic release pattern was reported for the 15-day study period with conduits B, C, and D. An escalating rapid-release phase was noted during the first week followed by a plateaued effect achieving a near zero-order release over the final week. Conduit B, having the NGF layer deposited just outside the PEC layer had the highest burst release, particularly during the rapid-release phase. Conduit D, having the NGF layers interposed between additional layers of PLGA, was more efficient in minimising burst release and significantly prolonging the delivery of NGF at the end of 15 days [19]. Using a similar concept of PEC formation, Xu and coworkers, 2011, developed a multilayered nerve conduit using a layer-by-layer electrostatic self-assembly technique to create a PDLLA/chondroitin sulphate/chitosan PEC of varying layers. NGF was immobilised onto the conduit via carbodiimide crosslinking. The nerve conduits were evaluated for their* in vitro* release kinetics of NGF and its effect on the potential of nerve regeneration across a 10 mm gap in the rat sciatic nerve model [27].

Despite a high initial burst release over the first day followed by a sharply plummeting release of NGF over the next day, the multilayered PEC system was successful in providing a zero-order profile with a sustained release of 1 ng NGF daily for over 50 days. Implantation of the conduit across a 10 mm sciatic nerve gap in the rat proved the conduit to be closely matched to the outcomes of the autograft when comparing the two in terms of myelination, axon diameter, and nerve conduction velocity over a period of 3 to 6 months [27]. PECs, particularly those fabricated from multilayered techniques, are being extensively investigated for the delivery of proteins and their use in tissue engineering applications [135, 136, 138, 139]. Besides presenting a milder method of immobilisation for delicate proteins, PECs offer improved physicomechanical strength with a minimised swelling and erosion propensity thereby prolonging bioactive release [138, 140]. To obtain various release kinetics, proteins can be deposited onto the surface of a preformed PEC layer, in between PEC multilayers or distributed within a PEC layer for a steadier release [138]. PECs have been used for the delivery of proteins and peptides for several controlled release applications and should be further studied for their use in delivering NTFs in peripheral nerve injury.

## 6. Combined Delivery of Neurotrophic Factors: Effects on Neuronal Regeneration

The simultaneous delivery of two or more NTFs from nerve conduits may be more beneficial in peripheral nerve regeneration than the delivery of a single NTF. Each NTF has a unique therapeutic mechanism of promoting regeneration of and sustaining neuronal cells. The codelivery of NTFs may enhance nerve regeneration by targeting and promoting various different pathways of neuronal growth and survival in addition to reducing the initial loading and daily doses of NTFs thereby providing perhaps a more cost-effective route in utilising NTFs. Schwann cells (SCs), forming the predominant component of glial cells in the peripheral nervous system, are known to express a number of receptors for ECM molecules and NTFs [41, 141]. It is proposed that NTFs exhibit their actions by binding to two receptor types, namely, the p75NTR receptor and the Trk class of receptors comprising TrkA (NGF), TrkB (BDNF), and TrkC (NT-3) located on SCs and neurons throughout the nervous system [33, 142, 143]. SCs have been reported to particularly express high levels of the p75NTR receptor which NTFs act on to modulate the migration, proliferation, and myelination capacities of SCs in peripheral nerves [144–146]. The binding of NTFs to the p75NTR receptor is thought to occur with a similar low affinity across all NTFs whereas binding activity to the Trk class of receptors is more specific [147, 148]. NGF, having particular affinity for TrkA, functions to promote the survival and maintenance of sensory neurons whereas binding to p75NTR receptor sites on SCs enhances myelination of axons [33, 149]. Similarly, the neurotrophin BDNF is reported to be a potent promoter of myelination via activation of the p75NTR receptors located on SCs whereas activation of the same receptors by NT-3 enhances SC migration. It was noted by researchers that although NT-3 had positive effects on SC migration, activation of SC-TrkC receptors inhibited myelination [146, 149, 150]. GDNF has been investigated for its actions in the promotion of motor neuron survival, SC migration, and the induction of myelination of small axons via activation of the Ret tyrosine kinase [149, 151–153]. In this regard, cautious NTF selection for combined therapy is necessary as the delivery of more than one NTF may prove to confer either synergistic or opposing biological activity. Furthermore, the activation of specific receptors and their resulting effects on the different cell types found in peripheral nerves must be taken into account.

In an interesting study by Madduri and coworkers, 2010, it was shown that GDNF-releasing nerve conduits promoted only axonal elongation in chicken embryonic DRG whereas a combined-release of GDNF and NGF enhanced axonal elongation in addition to the promotion of branching of the nerve tissue. It was noted, in DRG assays, that optimal growth occurred at doses of 1–10 ng/mL of GDNF or NGF but combined GDNF and NGF required a total reduced dose range of 0.1–1 ng/mL for an optimal growth response. Furthermore,* in vivo* studies across a 10 mm nerve gap showed significantly improved axonal outgrowth when using a combination of 40 ng GDNF and NGF each in comparison to 80 ng GDNF alone [52]. To enhance the synergistic effect of the GDNF/NGF combination, a variety of collagen and silk fibroin nerve conduits were designed for the extended and simultaneous release of the growth factors in a zero-order fashion [151].

The selection of NTFs for combined delivery to achieve a synergistic response in the enhancement of axonal regeneration must be carefully determined as not all NTFs may work synergistically for a heightened effect. Another investigation, using concentration gradients of NTFs, showed that combined concentrations of NGF and BDNF had no significant effect of synergism when evaluated for axonal growth response in DRG; however, a combination of NGF and NT-3 was shown to be successful in achieving synergism at concentrations of 80 ng/mL/mm each. The NGF/NT-3 combination of growth factors was more effective in axonal regeneration compared to a single dose of NGF of 133 ng/mL/mm concentration and capable of guiding axons over 12.5 mm distance as opposed to that of 7.5 mm, respectively [51].

## 7. Cell-Engineered Applications for Peripheral Nerve Regeneration

Another technique of delivering neurotrophic factors to injured peripheral nerves involves the exogenous culturing of support cells into the matrix of natural or synthetic-based conduits. These cells, such as, Schwann cells (SCs), mesenchymal, neural, and embryonic stem cells, interact via various molecular pathways to bring about peripheral nerve regeneration by providing substrates and molecules that partake of a pivotal regulatory function in axonal migration and proliferation [41, 154]. The purported positive interactions between support cells and peripheral nerves led to the inclusion of these cells into nerve conduits—a growing field of interest as an alternative to the nerve autograft.

Schwann cells, being the principal support cells of the peripheral nervous system, are capable of secreting neurotrophic factors in addition to providing an ECM-scaffold system and hence fulfilling the fundamental requirements for an environment sustainable of neural cell growth [155]. Several researchers have investigated SC transplantation and overexpression of genes encoding specific proteins as a promising outlook for peripheral nerve regeneration [35, 41, 155–157]. Studies have shown the regenerative potential of genetically modified SCs overexpressing fibroblast growth factor, in short and long gap sciatic nerve defects of 5 mm and 15 mm, respectively [158, 159]. A group of investigators designed isogenic NGF-transduced SCs for the supply of NGF immediately after nerve injury and during the early stages of nerve regeneration when endogenous neurotrophin levels are particularly low [35]. The overexpression of NGF from the transplanted SCs was able to provide long-term and increased delivery of the NTF for at least two weeks after induction of sciatic nerve injury. However, in the presence of proliferating SCs, bands of Büngner are formed which guide axonal regeneration and target tissue innervation from the proximal to the distal stump of the transected nerve thus enhancing nerve regeneration potential [41, 155]. Since, it is thought that SCs secrete a number of NTFs comprising of NFG, BDNF, and GDNF which act through different mechanisms to enhance and accomplish complete nerve regeneration, the incorporation of SCs may be considered a valuable addition in enhancing the functionality of nerve conduits [154, 155, 160]. The same group of researchers later developed a method for the regulation of GDNF expression using dendrimers and lentiviral transduction for the modification SCs paired with the administration of doxycycline for improved functional recovery in a rat nerve model [161]. Similarly, the forthcoming research focused on the ability of modified SCs to deliver GDNF for axonal regeneration [162, 163].

Although SCs are popular for the investigation of cell and gene-based therapy in neural regeneration strategies, neural and mesenchymal-derived stem cells have likewise shown significance in the ability to deliver NTFs such as BDNF, GDNF, and NGF [164–167]. Such cell-based delivery applications may offer several advantages in terms of providing suitable release kinetics for the delivery of multiple NTFs in therapeutically adequate quantities while the concerns of protein degradation and inactivation from manufacturing processes may be eradicated thereby ensuring complete bioactivity of these physiologically delivered NTFs [160, 168].

## 8. Conclusions

The therapeutic benefits offered by the use of neurotrophic growth factors for the application in enhancing regeneration and healing of transected and damaged peripheral nerves are noteworthy. The full potential of such potent growth factors can only be harvested if employed in a delivery system capable of precisely releasing adequate quantities of growth factor for a sufficient amount of time following the most desirable release kinetics, preferably zero-order release or a via a delivery system that offers a gradient-based release of NTFs. Burst release mechanisms must be further investigated so that minimal quantities of bioactives are released upon* in vivo* implantation of the nerve conduit resulting in potential hindrance of nerve growth. Furthermore, bioactivity of entrapped NTFs must be ascertained as fabrication procedures influence their ultimate therapeutic potency. In conjunction with* in vitro* release studies, animal models are valuable in assessing the effects and differences of the observed release kinetic profiles as similar release kinetics cannot be simply assumed to occur* in vivo*.* In vitro* studies cannot fully emulate the biological and physiological conditions present in the body especially after subjection of internal organs to injury. Hence, different studies on different types of nerve conduits may present with similar release kinetics compared to each other but have different regeneration effects on the regeneration potential on tissues. Although numerous new polymeric nerve conduits delivery systems have been proposed with several designs suggesting great promise in the significant improvement of peripheral nerve regeneration, further work must be carried out to smooth out concerns associated with release kinetics, ideal NTF selection, and dosing in accordance with tissue requirements, the length of treatment, and restoration of functional recovery.

## Figures and Tables

**Table 1:** Salient features of an ideal nerve conduit.

Conduit features and requirements	Importance and function	References
Mechanical attributes	(i) Should not bend, buckle, or kink postimplantation (ii) Be able to maintain structural integrity to support tissue growth	[2, 16, 24]
	(i) Overly pliable conduits are unable to withstand pressure from growing and surrounding tissues (ii) Rigid conduits may cause compression and damage to growing and surrounding tissues For example, blood vessels and nerve stumps (iii) Sufficient flexibility to withstand body movements, particularly at joint sites	[24, 25, 30]

Biodegradation rate	(i) Should degrade at a rate corresponding to tissue regeneration (ii) Premature disintegration removes provision of support structure (iii) Lengthy degradation may cause compression and inflammation of newly generated tissues	[30, 31]

Semipermeability	(i) Should allow exchange of oxygen and nutrients and elimination of waste products between internal and external environment of conduit (ii) Prevent infiltration of inflammatory cells and fibrotic tissue (iii) Prevent escape of neurotrophic growth factors secreted by damaged nerve stumps and from system	[16, 24, 30]

Physical and 3D guidance cues	(i) Cylindrical and channel-based structures for growth cone guidance (ii) Prevent axonal misdirection during regeneration towards distal nerve	[15, 24, 29]

Ability to deliver growth factors or Schwann cells	(i) Enhancement of functional recovery and axonal regeneration (ii) Important for neuronal survival and differentiation (iii) Schwann cells for support of axon regeneration and remyelination	[22, 30, 32–35]

Processing requirements	Maintenance of stability during handling, sterilization, storage, and surgical procedures	[24]

## Abbreviations

BDNF: Brain-derived neurotrophic factor
BSA: Bovine serum albumin
DHT: Dehydrothermal treatment
DRG: Dorsal root ganglia
EngNT: Engineered neural tissue
GDNF: Glial-derived neurotrophic factor
NGF: Nerve growth factor
NT-3: Neurotrophin-3
NTFs: Neurotrophic factors
PCL: Poly(caprolactone)
PDLLA: Poly(D,L-lactic acid)
PEAD: Poly(ethylene argininyl aspartate diglyceride)
PEC: Polyelectrolyte complex
PEG: Poly ethylene glycol
PLGA: Poly(lactic-co-glycolic) acid
PLLA: Poly(lactide)
PPE: Polyphosphoester
SC: Schwann cell
SH3: Src homology domain 3
STPP: Sodium tripolyphosphate.
